# Gut Microbiota and Serum Metabolic Signatures of High-Fat-Induced Bone Loss in Mice

**DOI:** 10.3389/fcimb.2021.788576

**Published:** 2021-12-22

**Authors:** Lingyun Lu, Mengjia Tang, Jiao Li, Ying Xie, Yujue Li, Jinwei Xie, Li Zhou, Yi Liu, Xijie Yu

**Affiliations:** ^1^ Department of Endocrinology and Metabolism, Laboratory of Endocrinology and Metabolism, Department of Integrated Traditional Chinese and Western Medicine, Rare Disease Center, West China Hospital, Sichuan University, Chengdu, China; ^2^ Department of Endocrinology and Metabolism, Laboratory of Endocrinology and Metabolism, Rare Disease Center, West China Hospital, Sichuan University, Chengdu, China; ^3^ Department of General Practice, West China Hospital, Sichuan University, Chengdu, China; ^4^ Department of Orthopaedic Surgery and National Clinical Research Center for Geriatrics, West China Hospital, Sichuan University, Chengdu, China; ^5^ Core Facilities of West China Hospital, West China Hospital, Sichuan University, Chengdu, China; ^6^ Department of Rheumatology and Immunology, Rare Disease Center, West China Hospital, Sichuan University, Chengdu, China

**Keywords:** osteoporosis, gut microbiota, metabolome, high-fat diet, bone loss

## Abstract

**Background:**

Accumulating evidence indicates that high-fat diet (HFD) is a controllable risk factor for osteoporosis, but the underlying mechanism remains to be elucidated. As a primary biological barrier for nutrient entry into the human body, the composition and function of gut microbiota (GM) can be altered rapidly by HFD, which may trigger abnormal bone metabolism. In the current study, we analyzed the signatures of GM and serum metabolomics in HFD-induced bone loss and explored the potential correlations of GM and serum metabolites on HFD-related bone loss.

**Methods:**

We conducted a mouse model with HFD-induced bone loss through a 12-week diet intervention. Micro-CT, Osmium-μCT, and histological analyses were used to observe bone microstructure and bone marrow adipose tissue. Quantitative Real-Time PCR was applied to analyze gene expression related to osteogenesis, adipogenesis, and osteoclastogenesis. Enzyme-linked immunosorbent assay was used to measure the biochemical markers of bone turnover. 16s rDNA sequencing was employed to analyze the abundance of GM, and UHPLC-MS/MS was used to identify serum metabolites. Correlation analysis was performed to explore the relationships among bone phenotypes, GM, and the metabolome.

**Results:**

HFD induced bone loss accompanied by bone marrow adipose tissue expansion and bone formation inhibition. In the HFD group, the relative abundance of *Firmicutes* was increased significantly, while *Bacteroidetes*, *Actinobacteria*, *Epsilonbacteraeota*, and *Patescibacteria* were decreased compared with the ND group. Association analysis showed that thirty-two bacterial genera were significantly related to bone volume per tissue volume (BV/TV). One hundred and forty-five serum metabolites were identified as differential metabolites associated with HFD intervention, which were significantly enriched in five pathways, such as purine metabolism, regulation of lipolysis in adipocyte and cGMP-PKG signaling pathway. Sixty-four diffiential metabolites were matched to the MS2 spectra; and ten of them were positively correlated with BV/TV and five were negatively correlated with BV/TV.

**Conclusions:**

These findings indicated that the alternations of GM and serum metabolites were related to HFD-induced bone loss, which might provide new insights into explain the occurrence and development of HFD-related osteoporosis. The regulatory effects of GM and metabolites associated with HFD on bone homeostasis required further exploration.

## Introduction

Osteoporosis (OP) is a common skeletal disease with pathological characteristics of decreased bone density, destroyed bone microstructure, and increased bone fragility (Zhu et al., 2021). Its occurrence and development are closely related to aging, menopause, nutritional deficiency or overnutrition, and a sedentary lifestyle (Pouresmaeili et al., 2018; Zhu and Zheng, 2021).

High-fat diet (HFD) is defined as a diet containing lipids that account for more than 30% of the total energy intake (Qiao et al., 2021). HFD has complicated correlations with bone metabolism. Traditionally, HFD is believed to be a skeletal protector because it causes weight gain, and high body weight is widely considered to be a protective factor for bone health (Felson et al., 1993). However, recent studies have found that excess dietary fat intake can disrupt bone remodeling, accelerate bone aging, and is an independent and controllable risk factor for osteoporosis (Kwon et al., 2015; Montalvany-Antonucci et al., 2018; Li et al., 2020).

Gut microbiota (GM) is a primary biological barrier for nutrient entry into the human body, abnormal perturbation of which leads to skeletal deterioration (Lu et al., 2021). The composition and function of GM can be altered rapidly by dietary nutrition (David et al., 2014; Aguirre et al., 2016); in turn, the absorption, utilization, and metabolism of dietary nutrition will be affected by the altered GM (Gentile and Weir, 2018). The adverse effect of HFD on GM is a key factor mediating bone homeostasis. HFD evokes intestinal inflammation and damages the intestinal mucosal barrier, following which intestinal microbes transfer into the circulatory system and affect bone metabolism (Qiao et al., 2021). It has been shown that the GM regulates HFD-induced metabolic stress and bone marrow niche function (Luo et al., 2015). Nowadays, research on the relationship between GM and HFD-related bone loss is still in the preliminary stage. Understanding the characteristics of GM and GM-derived metabolites under conditions of HFD-induced bone loss is very important, and this may be a new insight that will contribute to the development of osteoporosis prevention and treatment.

## Methods

### Experimental Animals

C57BL/6 male mice (7 weeks old) came from the Laboratory Animal Center of Sichuan University. Mice were housed in specific pathogen-free conditions (23 ± 1°C, 12/12 h light-dark cycle) and given free access to sterile food and autoclaved water ad libitum in cages. After feeding for one week under this condition, the mice were randomly divided into a high-fat diet (HFD) group and a normal diet (ND) group, with six mice in each group. The composition of ND and HFD from Beijing HFK Bioscience Corporation is shown in **Supplementary Table 1**. After a 12-week diet intervention, mice were fasted for 12 h at the end of the experiment and euthanized under general anesthesia. All animal procedures in our experiments were performed in strict accordance with the guidelines provided by the CPCSEA and *World Medical Association Declaration of Helsinki* on Ethical Principles and were approved by the Institutional Animal Care and Treatment Committee of Sichuan University in China (Permit number: 2020136A).

### Analysis of Bone Microstructure by Micro-CT

Femurs were isolated and immersed into fixative solution for micro-CT. A high-resolution micro-CT system (vivaCT80; Scanco Medical, Switzerland) was used to analyze the bone microstructure of trabecular bone of the distal femoral metaphysis. The scanner was set at a voltage of 55 kVp, a current of 145 μA, and a voxel size of 10 μm. Three-dimensional (3D) reconstruction and analysis were performed using Scanco software v.5.0. The domain of trabecular bone was manually profiled and interpolated with the contouring algorithm to choose a region of interest (ROI). One hundred contiguous cross-sectional slices from the growth plate of each femur were selected to analyze the volume and structure of trabecular bone. Parameters, including bone volume per tissue volume (BV/TV), trabecular number (Tb. N), trabecular thickness (Tb. Th), and trabecular spacing (Tb. Sp), were calculated.

### Histological Analysis

The tibias were removed from the soft tissue, fixed in 4% paraformaldehyde for 12 h and decalcified in 20% ethylenediaminetetraacetic acid (EDTA) solution at 37°C for 5–7 days until the tibias turned soft. The tibias were then dehydrated, embedded in paraffin, cut into 5 mm longitudinal sections, dried, stained with hematoxylin-eosin (H&E), and kept at room temperature.

### Quantification of Bone Marrow Fat

The bone marrow adipose tissue (BMAT) was quantified by osmium tetroxide staining with micro-CT scanning (Scheller et al., 2014). The tibias were fixed in 4% paraformaldehyde for 24 h, washed for 5 min, and decalcified in 20% EDTA solution at 37°C for 14 d. The decalcification solution was replaced every 3 d until the bones were pliable. A solution of potassium dichromate (750 μL 5%) and osmium tetroxide (750μL 2%) was added to 2 ml microtubes and 2–3 bones were soaked in each tube. The bones were immersed in the dye for 60 h at room temperature and then washed with a flow of distilled water for 2 h. Next, the bones were scanned by micro-CT with the parameters set at a voltage of 90 kVP, a current of 88 μA, and a voxel size of 50 μm. Image J software was used to analyze the proportion of osmium tetroxide staining area in the sagittal plane of the tibia, which represented the relative content of bone marrow fat.

### Gene Expression Analysis

Total RNA was extracted from the bone marrow and metaphysis according to the protocol provided by the manufacturer with TRIzol reagent (Invitrogen, Thermo Fisher Scientific, USA). RNA was reverse transcribed to cDNA with PrimeScript™ RT reagent Kit with gDNA Eraser (Takara, Japan), and transcripts were quantified by real-time PCR using SYBR Premix Ex Taq II (Takara, Japan). Primer sequences are summarized in **Supplementary Table 2**. The relative mRNA levels of target genes were normalized to β-actin, and the data were analyzed by the 2^-ΔΔCT^ method.

### Enzyme-Linked Immunosorbent Assay

Blood was collected from the retroorbital vein of mice after 10-hour fasting and centrifuged for 15 min at 3000 rpm to separate the serum. ELISA kits (MBBiology Biological, Jiangsu, China) were used for detecting serum P1NP and β-CTX levels.

### DNA Extraction from Fecal Samples, 16S rRNA Gene Amplification, and Sequencing

The fresh feces from non-fasting mice were collected in the way of spontaneous defecation. Fecal samples were collected in sterile microtubes and stored in liquid nitrogen. Fecal flora DNA was extracted using a HiPure Stool DNA extraction kit (Magen, Guangzhou, China). PCR reactions were performed in triplicate, in 50 μL mixtures containing 10 μL of 5 × Q5@ Reaction Buffer, 10 μL of 5 × Q5@ High GC Enhancer, 1.5 μL of 2.5 mM dNTPs, 1.5 μL of each primer (10 μM), 0.2 μL of Q5@ High-Fidelity DNA Polymerase, and 50 ng of template DNA (New England Biolabs, USA). The 16S rDNA target region of the ribosomal RNA gene was amplified by PCR (95°C for 5 min, followed by 30 cycles at 95°C for 1 min, 60°C for 1 min, 72°C for 1 min, and a final extension at 72°C for 7 min) using primers 341F/806R (341F: CCTACGGGNGGCWGCAG; 806R: GGACTACHVGGGTATCTAAT). Amplicons were extracted from 2% agarose gels and purified using an AxyPrep DNA Gel Extraction Kit (Axygen Biosciences, Union City, CA, USA) according to the manufacturer’s instructions and quantified using an ABI StepOnePlus Real-Time PCR System (Life Technologies, Foster City, USA). Purified amplicons were pooled in equimolar and paired-end sequenced (PE250) on an Illumina platform according to standard protocols.

### Microbial Community Analysis

High-quality clean reads were obtained by filtering low-quality raw data containing more than 10% of unknown nucleotides or less than 50% of bases with quality (Q-value)>20. Paired-end clean reads were merged as raw tags using FLSAH (version 1.2.11) with a minimum overlap of 10 bp and mismatch error rates of 2%. Noisy sequences of raw tags were filtered under specific filtering conditions (Bokulich et al., 2013) to obtain the high-quality clean tags. Then, the clean tags were clustered into operational taxonomic units (OTUs) of  ≥97% similarity using UPARSE pipeline (Edgar, 2013) (v.9.2.64). All chimeric tags were removed using the UCHIME algorithm (Edgar et al., 2011) and finally effective tags were obtained for further analysis. The tag sequence with highest abundance was selected as a representative sequence within each cluster. Taxonomy annotation and bioinformatics analysis, such as community composition, indicator species, Alpha diversity, Beta diversity, function prediction, and environmental factor, were carried out based on OTUs. Analyses were conducted by Gene Denovo Biotechnology Co. (Guangzhou, China). In brief, the representative OTU sequences were classified into organisms by a naive Bayesian model using RDP classifier (v.2.2) (Wang et al., 2007) based on the SILVA database (v.132) (Pruesse et al., 2007), with a confidence threshold value of 0.8. Abundance statistics for each classification were displayed using KRONA (v.2.6) (Ondov et al., 2011). Visualization of microbiome communities were performed with the dynamic real-time interactive online platform Omicsmart (http://www.omicsmart.com) or R project, utilizing the phyloseq package (McMurdie and Holmes, 2013).

### Serum Metabolites Extraction

Blood was collected from mice after 10-hour fasting and then centrifuged for 15 min at 3000 rpm to separate the serum. Serum samples (50 μL) were transferred to EP tubes. After adding 200 μL of extract solution (acetonitrile: methanol = 1:1, containing isotopically labelled internal standard mixture), the sample was vortexed for 30 s, sonicated for 10 min in an ice-water bath, and incubated for 1 h at -40°C to precipitate proteins. Then the sample was centrifuged at 12 000 rpm for 15 min at 4°C. The resulting supernatant was transferred to a fresh glass vial for analysis. The quality-control (QC) sample was prepared by mixing an equal aliquot of the supernatants from all samples.

### UHPLC-MS/MS Analysis of Serum Metabolites

LC-MS/MS analyses were performed using a UHPLC system (Vanquish, Thermo Fisher Scientific) with a UPLC BEH Amide column (2.1 mm × 100 mm, 1.7 μm) coupled to a Q Exactive HFX mass spectrometer (Orbitrap MS, Thermo). The mobile phase consisted of 25 mmol/L ammonium acetate and 25 mmol/L ammonium hydroxide in water (pH = 9.75) (A) and acetonitrile (B). The analysis was carried with elution gradient as follows: 0~0.5 min, 95% B; 0.5~7.0 min, 95%~65% B; 7.0~8.0 min, 65%~40% B; 8.0~9.0 min, 40% B; 9.0~9.1 min, 40%~95% B; and 9.1~12.0 min, 95% B. The column temperature was 30°C. The auto-sampler temperature was 4°C, and the injection volume was 2 μL. The QE HFX mass spectrometer was used for its ability to acquire MS/MS spectra on information-dependent acquisition (IDA) mode in the control of the acquisition software (Xcalibur, Thermo). In this mode, the acquisition software continuously evaluates the full scan MS spectrum. The ESI source conditions were set as follows: sheath gas flow rate as 50 Arb, Aux gas flow rate as 10 Arb, capillary temperature 320°C, full MS resolution as 60 000, MS/MS resolution as 7500, collision energy as 10/30/60 in NCE mode, and spray voltage as 3.5 kV (positive) or -3.2 kV (negative), respectively.

### Data Processing and Annotation of Serum Metabolome Data

The raw data were converted to the mzXML format using ProteoWizard and processed with an in-house program using R package based on XCMS (v.3.2), including retention time alignment, peak detection, and peak matching. Then an in-house MS2 database (BiotreeDB) was applied for metabolite annotation. The cutoff for annotation was set at 0.3. Orthogonal projection to latent structures-discriminant analysis (OPLS-DA) was applied in comparison groups using R package models (http://www.r-project.org/). A variable importance in projection (VIP) score of (O)PLS model was used to rank the metabolites that best distinguished between the two groups. VIP threshold was set to 1. Additionally, the T-test was also used as a univariate analysis for screening differential metabolites. The multiple testing correction was performed using the Benjamini-Hochberg false discovery rate (FDR) method for the differential metabolites to control the false positive rate. Those with a *q-*value (*p*-value adjusted by FDR)<0.05, VIP >1 and |log2 FC(HFD/ND)| > 1 were considered as differential metabolites between the two groups. Metabolites were mapped to KEGG metabolic pathways for pathway analysis and enrichment analysis (Kanehisa et al., 2017). Pathway enrichment analysis identified significantly enriched metabolic pathways or signal transduction pathways in differential metabolites, making a comparison with the whole background. Pathways meeting the condition *q-*value <0.05 were defined as significantly enriched pathways in differential metabolites.

### Statistical Analysis

The statistical significance of bone mass and biochemical indexes was calculated using Student’s t-test or one-way analysis of variance (ANOVA) in GraphPad Prism (GraphPad Software Inc., USA, v.6.0). Spearman rank correlation was used for association analysis of 16s microbiome, metabolome and bone phenotypes. Statistical significance was indicated as follows: *P < 0.05, **P < 0.01, ***P < 0.001.

## Results

### High-Fat Diet Induced Obesity and Bone Loss in Mice

**Figure 1:**
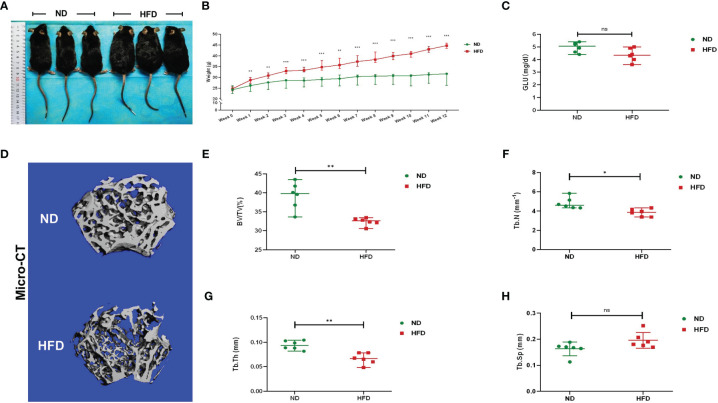
High-fat diet induced obesity and bone loss in mice. **(A)** Photographs of mice in each group after a 12-week dietary intervention of ND or HFD. **(B)** Body weight curves of mice after dietary intervention (n = 6). **(C)** Fasting blood glucose of the two groups (n = 6). **(D)** Representative images for micro-CT 3D reconstruction (scale bar = 100 mm). **(E–G)** Trabecular bone parameters at the distal femoral metaphysis, including BV/TV, Tb. N, Tb. Th, and Tb. Sp after ND or HFD treatment (n = 6). Data were expressed as mean ± SD. *P < 0.05, **P < 0.01, ***P < 0.001; ns, no significance.

Compared with ND, the weight of HFD mice was increased significantly after the 12-week diet intervention. At the end of the 12^th^ week, the mean weight of HFD mice was 44.61 g, 44.89% higher than the ND mice (**Figures 1A, B**). There was no significant difference in fasting blood glucose between the two groups (**Figure 1C**). Micro-CT was applied for analyzing bone mass and bone microstructure. Compared with ND, the bone microstructure of the distal femoral metaphysis in HFD mice was visibly destroyed (**Figure 1D**). There were distinct reductions of BV/TV, Tb.N, and Tb.Th (**Figures 1E–G**). These results indicated that HFD led to obesity and trabecular bone loss.

### HFD Promoted Bone Marrow Adipose Tissue Expansion and Inhibited Bone Formation

**Figure 2:**
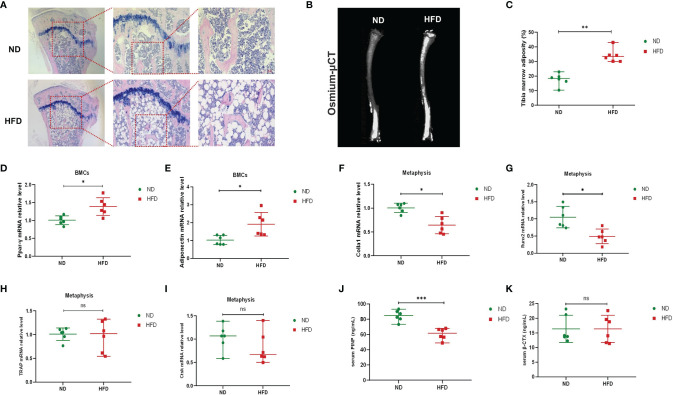
High-fat diet increased bone marrow fat, promoted adipogenesis, and inhibited osteogenesis. **(A)** Histopathological analysis on bone sections from tibia stained with H&E after ND or HFD treatment. **(B)** Representative images of BMAT from full-length tibial sagittal plane of ND or HFD mice by micro-CT. **(C)** Quantification of the osmium density in tibia sagittal plane (n = 6). **(D, E)** mRNA levels of adipogenic genes in BMCs of ND or HFD mice (n = 6). **(F, G)** mRNA levels of osteoblastic genes in BMCs of ND or HFD mice (n = 6). **(H, I)** mRNA levels of osteoclastic genes in the metaphysis of ND or HFD mice (n = 6). **(J, K)** Serum levels of bone turnover biomarkers (n = 6). Data were expressed as mean ± SD. *P < 0.05, **P < 0.01, ***P < 0.001, ns, no significance.

Previous studies have shown that in addition to peripheral fat, HFD could also increase bone marrow fat and that the unbalanced dynamic equilibrium of osteogenesis and adipogenesis was key to HFD-induced bone loss (Styner et al., 2014; Li et al., 2020). We investigated the content of bone marrow fat *via* H&E staining and osmium-μCT. Compared with ND mice, adipocytes in proximal tibia and the osmium signal of HFD mice were increased remarkably (**Figures 2A–C**). The relative expressions of adipogenic gene expressions (Ppar-γ and Adipoq) were increased in BMCs (**Figures 2D, E**) of HFD mice. Meanwhile, the genes associated with osteogenic differentiation (Colla1 and Runx2) were decreased in the metaphysis of HFD mice (**Figures 2F, G**), while those related to osteoclastic differentiation (Trap and Ctsk) showed no difference between the two groups (**Figures 2H, I**). Furthermore, serum markers of bone turnover showed that HFD increased P1NP (**Figure 2J**) and had no significant effect on β-CTX (**Figure 2K**). These results demonstrated that HFD might promote the accumulation of bone marrow adipose tissue, blunt bone formation and aggravate bone loss.

### HFD Altered the Composition of Gut Microbiota

**Figure 3:**
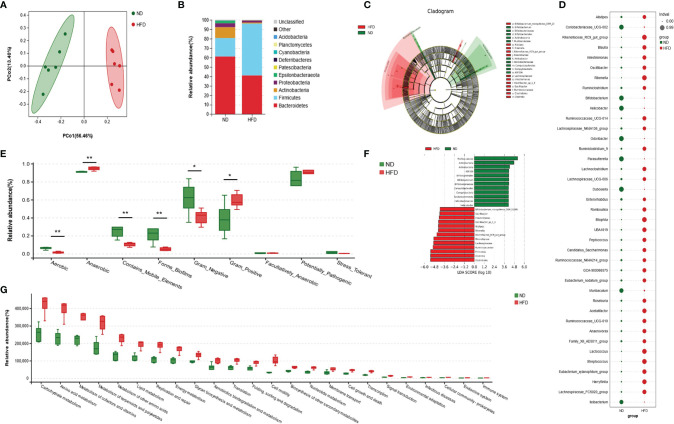
High-fat diet altered the composition and function of gut microbiota. **(A)** Principal coordinate analysis (PCoA) of gut microbiota (GM) in ND-fed and HFD-fed mice (n = 6). **(B)** The relative abundance of GM at the phylum-level in both groups (n = 6). **(C, F)** Cladogram showing the most differentially abundant taxa identified by linear discriminant analysis effect size (LEfSe). Red indicates clades enriched in the HFD group, and green indicates clades enriched in the ND group. Genera meeting a linear discriminant analysis (LDA) score threshold > 4 are shown in **Figure 3F** (n = 6). **(D)** Indicator analysis through calculating indicator value (IndVal). The greater the IndVal, the more likely the genus is to become the indicator for the group (n = 6). **(E)** Bugbase prediction of GM phenotypes for both groups (n = 6), *P < 0.05, **P < 0.01. **(F)** Functional enrichment analysis of GM based Kyoto Encyclopedia of Genes and Genomes (KEGG) pathway. The KEGG pathways of level 2 (*P*<0.05) are listed (n = 6).

According to principal coordinate analysis (PCoA), gut microbiota of the two groups showed a good separation, indicating a significant alteration of GM composition in HFD mice (**Figure 3A**). A composition analysis was performed at different levels (phylum, class, order, family, genus, and species); the phylum-level analysis is shown in **Figure 3B** and the results of other levels are listed in **Supplementary Figure 1**. The relative abundance of *Firmicutes* was increased significantly, while *Bacteroidetes, Actinobacteria*, *Epsilonbacteraeota*, and *Patescibacteria* were decreased in the HFD group compared with the ND group. To display indicator species visually, we performed an LDA effect size analysis (LEfSe) with LDA fold = 4; the relationship between different microbiota from the phylum level to the genus level is shown in the cladogram in **Figures 3C, F**. Compared with the ND group, *Bifidobacterium_mongoliense_DSM_21395*, *Oscillibacter*, *Intestinimonas*, *Oscillibacter_sp1_3*, *Alistipes*, *Rikenella*, *Rikenellaceae_RC9_gut_group*, *Rikenellaceae*, *Lachnospiraceae*, *Ruminococcaceae*, *Firmicutes*, *Clostridia*, and *Clostridiales* were increased in the HFD group; while *Murbaculaceae*, *Actinobacteria*, *Actinobacteria*, *ASF356*, *Bifidobacteriales*, *Bifidobacterium*, *Bifidobacteriaceae*, *Campylobacterales*, *Campylobacteria*, and *Epsilonbacteraeota* were decreased. We further employed indicator analysis and calculated indicator value (IndVal; **Figure 3D**). Thirty-eight indicators at the genus level were found in this method, and the genera with the most statistically significant differences were *GCA-900066575*, *Odoribacter*, *Parasutterella*, *Rikenella*, *Roseburia*, *Ruminococcaceae_UCG-014*, *UBA1819*, *Bilophila*, *Eubacterium_xylanophilum_group*, *Oscillibacter*, *Peptococcus*, and *Streptococcus*. A comparison of bacterial phenotype classification found that the intestinal flora belonging to Aerobic, Contains_Mobile_Elements, Forms_Biofilms, and Gram_Negative were reduced, while Anaerobic and Gram_Positive were increased in the HFD group (**Figure 3E**). Kyoto Encyclopedia of Genes and Genomes (KEGG) was used to annotate the function of the flora. Data showed that many metabolic pathways were activated in the HFD group, including carbohydrate, amino acids, terpenoids, polyketides, and lipids (**Figure 3G**). Further analysis of the enriched pathways found that, besides carbohydrate metabolism, HFD-altered GM mainly responded to amino acid metabolism, including increased histidine, glutathione, D-glutamine, D-glutamate, valine, leucine, and isoleucine (**Supplementary Figure 2**). In addition, the enrichment of disease pathways showed that the functions of HFD-altered GM were more concentrated in the infectious diseases, the endocrine system, and immune system (**Figure 3G**). This might have been evoked by the peptidoglycan of gram-positive bacteria *via* the NOD-like receptor (NLR) pathway, as the functions of GM were enriched in peptidoglycan biosynthesis and NLR signaling pathways (**Supplementary Figure 2**).

### HFD Changed the Serum Metabolome

**Figure 4:**
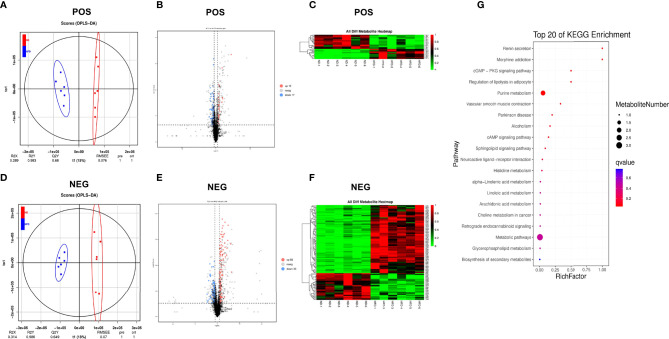
High-fat diet changed the serum metabolome. **(A, D)** OPLS-DA scores plot of serum metabolite profiling between the ND and HFD groups in both the positive and negative modes. **(B, E)** Volcano plot analysis of serum metabolites (VIP > 1,|P(corr)| > 0.5, jackknifed 95% confidence intervals). **(C, F)** Heat maps of serum metabolites of the two groups based on the linear transformation of the raw data (the interval of [0,1]). **(G)** The bubble diagram of the top 20 KEGG enriched pathways.

Orthogonal projection to latent structures-discriminant analysis (OPLS-DA) was performed to identify different fecal metabolites between the two groups (**Figures 4A, D**), and the models were validated *via k*-fold cross-validation (**Figures 4A, D**) and permutation test (**Supplementary Figure 3**). Compared with the ND group, a total of 29 metabolites in positive ion mode (12 upregulated and 17 downregulated) and 116 metabolites in negative ion mode (83 upregulated and 33 downregulated) were confirmed (**Figures 4B, E**). Cluster analysis of normalized data showed that there were significant cumulative differences in metabolites between the two groups (**Figures 4C, F**). Among these metabolites, 64 metabolites matched to the MS2 spectra were identified and listed in **Supplementary Tables 3**, **4**. KEGG topology analysis showed that the differential serum metabolites induced by HFD were mainly concentrated in five pathways (*q*-value < 0.05), such as the purine metabolism, regulation of lipolysis in adipocyte and cGMP-PKG signaling pathway (**Figure 4G**). Adenosine, one of the differential metabolites, was a common component of the above enriched pathways (**Supplementary Table 5**).

### Association Analysis of Gut Microbiota, Serum Metabolome and Bone Phenotypes

**Figure 5:**
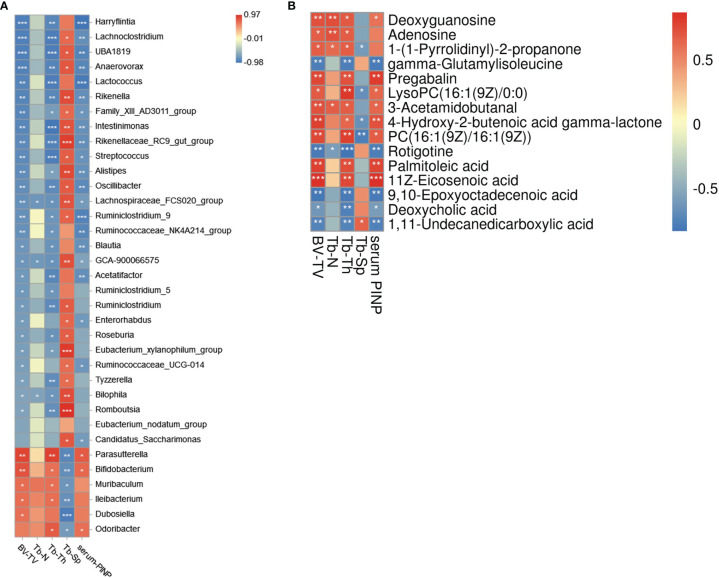
Association analysis of gut microbiota, bone phenotypes, and serum metabolome. **(A)** The heat map of Spearman rank correlation coefficients between the GM and bone phenotypes (|r|>0.6). **(B)** The heat map of Spearman rank correlation coefficients between the serum metabolites and bone phenotypes. *P < 0.05, **P < 0.01, ***P < 0.001.

Correlation analysis of bacteria genera and bone phenotypes was plotted in a heatmap (**Figure 5A**) (|r| > 0.6). Among the bacterial genera that were significantly related to BV/TV (|r| > 0.6, *P* > 0.05), a total of thirty-two genera belonged to differential genera for the two groups, such as *Parasutterella* (r = 0.867), *Bifidobacterium* (r=0.800), *Harryflintia* (r=-0.917), *Lachnoclostridium* (r=-0.917), UBA1819 (r=-0.917), *Anaerovorax* (r=-0.9), *Lactococcus* (r=-0.883) and *Rikenella* (r=-0.883; **Figure 5A**). Nineteen of twenty-seven genera negatively correlated with BV/TV belonged to *Clostridiales*, among which seven were *Lachnospiraceae* and eight were *Ruminococcaceae*. ROC curves of some differential bacteria correlated to BV/TV (|r| ≥ 0.8, *P* > 0.05) were shown in **Supplementary Figure 4**.

Correlation analysis of serum metabolites and bone phenotypes is shown in **Figure 5B** (|r| > 0.6, *P* > 0.05). BV/TV was positively related to adenosine, deoxyguanosine, palmitoleic acid, 1-(1-pyrrolidinyl)-2-propanone, 4-hydroxy-2-2butenoic acid gamma-lactone, 3-acetamidobutanal, 11z-eicosenoic acid, etc.; and negatively related to gamma−glutamylisoleucine, deoxycholic acid (DCA), 1,11−undecanedicarboxylic acid, etc. In the current study, we found HFD led to a significant decrease of adenosine and deoxyguanosine, the critical components of the purine metabolism pathway, which have been demonstrated to have bioactivities for bone metabolism. ROC curves of differential metabolites correlated to BV/TV (|r| ≥ 0.6, *P* > 0.05) were shown in **Supplementary Figure 5**.

### Association Analysis of Serum Metabolome and Gut Microbiota

**Figure 6:**
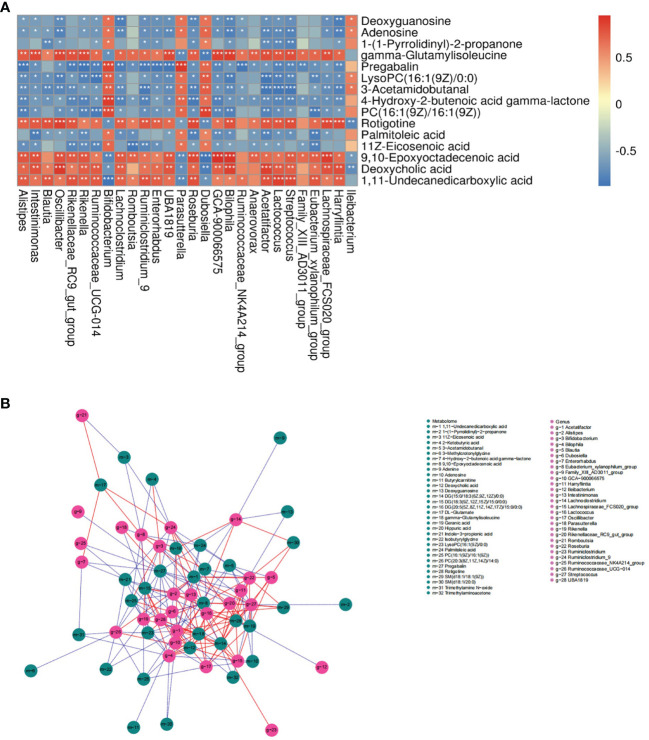
Association analysis of serum metabolome and gut microbiota. **(A)** Heat map of correlation between differentially expressed bacterial genera and metabolites in the ND and HFD group (|r|>0.6). *P < 0.05, **P < 0.01, ***P < 0.001. **(B)** Network map of the top 200 correlations between bacterial genera and serum metabolites related to bone phenotypes (|r|>0.5). A solid red line indicates a positive correlation; a dashed blue line indicates a negative correlation.

The correlation of serum metabolome and gut microbiota is shown in **Figure 6A** and the analysis of bone phenotype-related bacterial genera and metabolites is displayed in **Figure 6B**. Data showed multiple correlations between serum metabolites and bacterial genera. For example, adenosine and deoxyguanosine were positively related to *Bifidobacterium*, *Parasutterella*, *Dubosiella* and *Ileibacterium*; and negatively related to *Oscillibacter*, *Lachnoclostridium*, *Harryflintia*, *Blautia*, etc. *Bifidobacterium*, a group of bacteria known to have beneficial effects on bone health, had positive relations with adenosine, deoxyguanosine, 1-(1-pyrrolidinyl)-2-propanone, LysoPC(16:1(9Z)/0:0), 3-acetamidobutanal, 4-hydroxy-2-2butenoic acid gamma-lactone, PC(16:1(9Z)/16:1(9Z)), palmitoleic acid and 11z-eicosenoic acid; and negative relations with gamma−glutamylisoleucine, deoxycholic acid (DCA), 1,11−undecanedicarboxylic acid. From the perspective of known metabolites with bone bioactivity, HFD-induced bone loss might be associated with decreased adenosine.

## Discussion

GM is an important biological defense in the intestine, which can prevent the invasion of pathogenic microorganisms and maintain the physiological balance of the host. GM is an essential mediator for the process of high-fat-induced bone loss and its abnormal change can alter the bone niche microenvironment (Luo et al., 2015). Some previous studies have explored the effects of GM as a whole on bone metabolism related to HFD (McCabe et al., 2019; Zhang et al., 2021). However, detailed characteristic analysis of GM and the metabolome has not been conducted, which may contribute to the identification of novel biomarkers and therapeutic targets. In this study, we applied a multiomics correlation network approach to analyze the characteristics and relationships between the microbiome and serum metabolome in the development of high-fat-induced bone loss.

In the 16s rDNA sequencing analysis, an increase of *Firmicutes/Bacteroidetes* (F/B) ratio was found in the HFD group, which was also observed in ovariectomized mice with bone loss (Wen et al., 2020; Xie et al., 2020) and people with primary osteoporosis (Wang et al., 2017). Previous studies addressing obesity or nonalcoholic fatty liver disease have also demonstrated that HFD can induce an increase in F/B ratio (Li et al., 2021), which is closely related to intestinal immune inflammation (Zhao et al., 2021). In addition, HFD also led to a decrease in the abundance of SCFA-producing bacteria, such as *Bacteroides*, which exerts a bone protection effect *via* both promoting osteoblast differentiation (Chen et al., 2007) and inhibiting osteoclast differentiation (Lucas et al., 2018). A relatively high abundance of gram_positive bacteria and a relatively low abundance of gram_negative bacteria were observed in the HFD group, and GM function was significantly enriched in the peptidoglycan biosynthesis pathway. It is common knowledge that gram_positive bacteria includes *Firmicutes*. The peptidoglycan in their cell walls, which is the ligand for NLR, makes them the critical factor that activates the immune response (Bersch et al., 2021). Peptidoglycan-induced bone loss has been verified in a previous study (Dusad et al., 2013). Overall, it is speculated that HFD may activate the NLR signaling pathway through peptidoglycans in the gram_positive bacteria, and contribute to bone loss.

In the analysis of correlation between bacterial genera and bone phenotypes, *Bifidobacterium*, *Parasutterella*, *Odoribacter*, *Muribaculum*, *Ileibacterium* and *Dubosiella* were positively related to BV/TV (|r| > 0.6), which were significantly decreased in the HFD group. *Bifidobacterium* are widely known to be beneficial for bone growth and development (Whisner and Castillo, 2018), which can normalize gut barrier function and reduce pro-inflammatory cytokine levels (Madsen et al., 2001; Srutkova et al., 2015). *Parasutterella* was initially defined as a core component of the human and mouse GM that can contribute to host health in 2019 (Ju et al., 2019). The key role of *Parasutterella* is to enhance the catabolism of tryptophan, tyrosine, and bile acid components (Ju et al., 2019), which are potentially related to bone metabolism (Lu et al., 2021). A recent study has identified a strain of *Odoribacter* that protects against colitis and colorectal cancer, and demonstrated a crucial role in inducing immunosuppressive Th17 cells (Xing et al., 2021). As a hotspot of osteoimmunology-related research, the relationship between Th17 cells and bone metabolism is also being gradually explored (Tang et al., 2020). Additionally, the abundance of *Oscillibacter* and *Alistipes* has been evaluated in HFD-fed mice, and a similar change was observed in aging mice (van Beek et al., 2018). HFD can accelerate the senescence of BMSCs (Li et al., 2020); whether the above two aging-related bacteria are critical in this process is worth further investigation.

Metabolites bridge the distance between the GM and skeletal system (Zaiss et al., 2019). Various GM-derived metabolites have displayed bone metabolism-related bioactivities (Lu et al., 2021). Purine metabolism is one of the metabolic pathways enriched by differential metabolites after HFD (**Figure 4G**). Purine cascade contributes to maintain the differentiation capacity of MSCs into mature and functional osteoblasts (Noronha-Matos and Correia-de-Sá, 2016). Adenosine is an important part of this pathway and plays a key role in maintaining bone health *via* activation of specific cell surface G protein coupled receptors (A1, A2A, A2B, and A3), and proteins (Mediero and Cronstein, 2013; Hoque et al., 2021). The regulation of adenosine on both osteoclasts and osteoblasts varies with the dose. In physiological conditions, adenosine of relatively low concentration activates A1 receptor (A1R) and then promotes osteoclast differentiation through RANKL-induced expression of the transcription factors NFATc1 and c-fos (Kara et al., 2010; He and Cronstein, 2012). As the concentration of adenosine increases, A2AR is activated to inhibit the secretion of MCSF and RANKL and decrease pro-inflammatory cytokines (IL-1β and TNF-α), leading to the suppression of osteoclastogenesis (Mediero et al., 2012). On the contrary, A1R activated in BMSCs can inhibit osteoblast differentiation, while A2AR can induce osteoblastogenesis in a cAMP/PKA-dependent mechanism (Russell et al., 2007) and A2BR can promote bone formation by increasing the expression of osteoblast-related genes Runx2 and ALP (Katebi et al., 2009; Carroll et al., 2012). In the current study, compared with the control (considered as a physiological state), adenosine was significantly reduced after HFD and therefore might fail to activate A2AR and A2BR, which would theoretically result in bone loss owing to decreased osteogenic differentiation and enhanced osteoclast differentiation. This was partially consistent with the results of bone phenotypes.

The alteration of GM induced by HFD not only enhanced carbohydrate and lipid metabolism, but also evoked the metabolism of several amino acids (**Figure 3G**). Circulating amino acids and the metabolites play important roles in bone metabolism (Lu et al., 2021), such as tryptophan, hydroxyproline, cysteine, proline, and glutamine (You et al., 2014; Qi et al., 2016; Miyamoto et al., 2017; Zhao et al., 2018; Su et al., 2019). For instance, typtophan metabolites (indoles, indole derivatives, and kynurenine) have dual regulatory effects on both osteoclastogenesis and osteoblastogenesis *via* aryl hydrocarbon receptor-mediated NFATc1, ERK, and p38 MAPK pathways (Eisa et al., 2020; Liu et al., 2020a; Liu et al., 2020b). Meanwhile, glutamic acid metabolite (γ-aminobutyric acid) can stimulate osteoblastogenesis by upregulating osteogenesis-related genes *via* activating GABAB receptors and decreasing inflammatory cytokines (IL-6 and CRP) and reactive oxygen species (Muhammad et al., 2013). In our study, the serum indole-derived indole-propionic acid (IPA) decreased in mice after HFD, which was consistent with the results of Behera et al. (Behera et al., 2021). It has been demonstrated that IPA promoted osteogenic differentiation by enhancing mitochondrial transcription activator Tfam *via* increased binding of histone demethylase Kdm6b and decreased binding of H3K27me3 to the Tfam promoter (Behera et al., 2021).

In addition to dietary nutrition, GM can also modify host metabolites, such as bile acids (Ridlon et al., 2014). Bile acids are synthesized and secreted by liver cells, 95% of which enter the liver-gut axis, and a few are metabolized into secondary bile acids by GM, such as lithocholic acid (LCA) and deoxycholic acid (DCA). In the current study, serum DCA was increased in HFD-fed mice, which is consistent with a recent study (Wang et al., 2020). However, the opposite change in DCA was observed in mice with OVX-induced bone loss (Wen et al., 2020); therefore the supplementation of DCA may provide a favorable effect in protecting bone loss due to estrogen deficiency (Ahn et al., 2020). It is speculated that DCA is not responsible for HFD-induced bone loss, and the effect of DCA on bone metabolism may be sex- or hormone-dependent.

This study had some limitations. Although the correlations of GM, serum metabolites, and HFD-related bone metabolism were explored for the first time in the current study, the causal relationship among the three and the regulatory effects of GM and metabolites on bone homeostasis were not clarified. For example, we found that gamma-Glutamylisoleucine (γ-Glu-Ile), a dipeptide composed of gamma-glutamate and isoleucine, was significantly increased after HFD, which was negatively correlated to BV/TV. Whether γ-Glu-Ile or other metabolites play physiological and pathological roles in the process of HFD-induced bone loss needs to be further explored. Furthermore, the sample size of this study was limited. Therefore, the differential bacteria and metabolites cannot be described as biomarkers for HFD-induced bone loss. Biomarkers were defined as “a characteristic that is objectively measured and evaluated as an indicator of normal biological processes, pathogenic processes, or pharmacologic responses to a therapeutic intervention” by Biomarkers Definitions Working Group at the National Institutes of Health (NIH) in 2001 (Biomarkers Definitions Working Group, 2001). High quality of biomarkers required the support of rigorous studies with adequate sample size (Pepe et al., 2015). Advances in high throughput technologies like next generation sequencing and mass spectrometry have made it possible for tens of thousands of biomarker candidates to be efficiently detected. However, massive amounts of data can cause combinatorial explosions, which means a sufficiently reliable predictor may be not only be a single variable, but also one of thousands of biomarker combinations. This implies a high risk of random associations. In order to solve the above problems, studies with large sample size are required, but it might not be feasible due to high costs and ethical reasons. Therefore, pilot studies with small sample size can act as an alternative (Al-Mekhlafi et al., 2020). Such pilot studies aim to discover the potential for a promising biomarker rather than to identify and validate the ultimate one. These results provide the evidence for whether an enlarged study is worthwhile, and can even be used as the basis for sample size calculation in the upcoming study (Al-Mekhlafi et al., 2020). Therefore, due to the limited sample size and the animal model, the results of this study can only provide some references to explore the mechanism of HFD-related osteoporosis, which need to be validated in studies with large sample size.

## Conclusion

Our study demonstrated that there weas a close correlation between GM and serum metabolites in HFD-induced bone loss. The alteration of GM composition and function caused by HFD may disrupt bone homeostasis *via* the “gut microbiota-metabolites-bone” axis. These findings provide new insights to explore the mechanism of HFD-related osteoporosis in the future.

## Data Availability Statement

The datasets presented in this study can be found in online repositories. The names of the repository/repositories and accession number(s) can be found below: sequencing - NCBI [accession: PRJNA778098]; metabolome data - CNSA [accession: CNP0002359].

## Ethics Statement

The animal study was reviewed and approved by Institutional Animal Care and Treatment Committee of Sichuan University in China.

## Author Contributions

XY designed this research. LL, MT, JL, YX, YJL, JX, and LZ were responsible for the experiments. Among them, LL, MT, JL, YJL and JX, were in charge of the animal experiments, cellular experiments, and molecular experiments. YX and LZ were mainly responsible for the histopathological part. LL, MT, JL, YL and XY were responsible for the revision of the whole article. All authors contributed to the article and approved the submitted version.

## Funding

This work was supported by the National Natural Science Foundation of China (No. 81770875, No. 81902246), the Key Research and Development Project of Science and Technology Department of Sichuan Province (2020YFS0142), the Health and Family Planning Commission of Sichuan Province (No. 19PJ096), the Post-Doctoral Research Project, West China Hospital, Sichuan University (No. 19HXBH053, No. 2020HXBH153), the Sichuan University (No.2018SCUH0093), and the 1.3.5 Project for Disciplines of Excellence, West China Hospital, Sichuan University (2020HXFH008, ZYJC18003).

## Conflict of Interest

The authors declare that the research was conducted in the absence of any commercial or financial relationships that could be construed as a potential conflict of interest.

## Publisher’s Note

All claims expressed in this article are solely those of the authors and do not necessarily represent those of their affiliated organizations, or those of the publisher, the editors and the reviewers. Any product that may be evaluated in this article, or claim that may be made by its manufacturer, is not guaranteed or endorsed by the publisher.

## References

[B1] Aguirre M. Eck A. Koenen M. E. Savelkoul P. H. Budding A. E. Venema K. (2016). Diet Drives Quick Changes in the Metabolic Activity and Composition of Human Gut Microbiota in a Validated *In Vitro* Gut Model. Res. Microbiol. 167 (2), 114–125. doi: 10.1016/j.resmic.2015.09.006 26499094

[B2] Ahn T. K. Kim K. T. Joshi H. P. Park K. H. Kyung J. W. Choi U. Y. (2020). Therapeutic Potential of Tauroursodeoxycholic Acid for the Treatment of Osteoporosis. Int. J. Mol. Sci. 21 (12), 4274. doi: 10.3390/ijms21124274 PMC7349164 32560070

[B3] Al-Mekhlafi A. Becker T. Klawonn F. (2020). Sample Size and Performance Estimation for Biomarker Combinations Based on Pilot Studies With Small Sample Sizes. Commun. Stat. Theory Methods 1–15. doi: 10.1080/03610926.2020.1843053

[B4] Behera J. Ison J. Voor M. J. Tyagi N. (2021). Probiotics Stimulate Bone Formation in Obese Mice via Histone Methylations. Theranostics 11 (17), 8605–8623. doi: 10.7150/thno.63749 34373761 PMC8344023

[B5] Bersch K. L. DeMeester K. E. Zagani R. Chen S. Wodzanowski K. A. Liu S. (2021). Bacterial Peptidoglycan Fragments Differentially Regulate Innate Immune Signaling. ACS Cent. Sci. 7 (4), 688–696. doi: 10.1021/acscentsci.1c00200 34056099 PMC8155477

[B6] Biomarkers Definitions Working Group (2001). Biomarkers and Surrogate Endpoints: Preferred Definitions and Conceptual Framework. Clin. Pharmacol. Ther. 69 (3), 89–95. doi: 10.1067/mcp.2001.113989 11240971

[B7] Bokulich N. A. Subramanian S. Faith J. J. Gevers D. Gordon J. I. Knight R. (2013). Quality-Filtering Vastly Improves Diversity Estimates From Illumina Amplicon Sequencing. Nat. Methods 10 (1), 57–59. doi: 10.1038/nmeth.2276 23202435 PMC3531572

[B8] Carroll S. H. Wigner N. A. Kulkarni N. Johnston-Cox H. Gerstenfeld L. C. Ravid K. (2012). A2B Adenosine Receptor Promotes Mesenchymal Stem Cell Differentiation to Osteoblasts and Bone Formation *In Vivo* . J. Biol. Chem. 287 (19), 15718–15727. doi: 10.1074/jbc.M112.344994 22403399 PMC3346096

[B9] Chen T. H. Chen W. M. Hsu K. H. Kuo C. D. Hung S. C. (2007). Sodium Butyrate Activates ERK to Regulate Differentiation of Mesenchymal Stem Cells. Biochem. Biophys. Res. Commun. 355 (4), 913–918. doi: 10.1016/j.bbrc.2007.02.057 17331472

[B10] David L. A. Maurice C. F. Carmody R. N. Gootenberg D. B. Button J. E. Wolfe B. E. (2014). Diet Rapidly and Reproducibly Alters the Human Gut Microbiome. Nature 505 (7484), 559–563. doi: 10.1038/nature12820 24336217 PMC3957428

[B11] Dusad A. Thiele G. M. Klassen L. W. Gleason A. M. Bauer C. Mikuls T. R. (2013). Organic Dust, Lipopolysaccharide, and Peptidoglycan Inhalant Exposures Result in Bone Loss/Disease. Am. J. Respir. Cell Mol. Biol. 49 (5), 829–836. doi: 10.1165/rcmb.2013-0178OC 23782057 PMC3931104

[B12] Edgar R. C. (2013). UPARSE: Highly Accurate OTU Sequences From Microbial Amplicon Reads. Nat. Methods 10 (10), 996–998. doi: 10.1038/nmeth.2604 23955772

[B13] Edgar R. C. Haas B. J. Clemente J. C. Quince C. Knight R. (2011). UCHIME Improves Sensitivity and Speed of Chimera Detection. Bioinformatics 27 (16), 2194–2200. doi: 10.1093/bioinformatics/btr381 21700674 PMC3150044

[B14] Eisa N. H. Reddy S. V. Elmansi A. M. Kondrikova G. Kondrikov D. Shi X. M. (2020). Kynurenine Promotes RANKL-Induced Osteoclastogenesis *In Vitro* by Activating the Aryl Hydrocarbon Receptor Pathway. Int. J. Mol. Sci. 21 (21), 7931. doi: 10.3390/ijms21217931 PMC7662708 33114603

[B15] Felson D. T. Zhang Y. Hannan M. T. Anderson J. J. (1993). Effects of Weight and Body Mass Index on Bone Mineral Density in Men and Women: The Framingham Study. J. Bone Miner. Res. 8 (5), 567–573. doi: 10.1002/jbmr.5650080507 8511983

[B16] Gentile C. L. Weir T. L. (2018). The Gut Microbiota at the Intersection of Diet and Human Health. Science (New York N.Y.) 362 (6416), 776–780. doi: 10.1126/science.aau5812 PMC13264711 30442802

[B17] He W. Cronstein B. N. (2012). Adenosine A1 Receptor Regulates Osteoclast Formation by Altering TRAF6/TAK1 Signaling. Purinergic Signal 8 (2), 327–337. doi: 10.1007/s11302-012-9292-9 22311477 PMC3350593

[B18] Hoque J. Shih Y. V. Zeng Y. Newman H. Sangaj N. Arjunji N. (2021). Bone Targeting Nanocarrier-Assisted Delivery of Adenosine to Combat Osteoporotic Bone Loss. Biomaterials 273, 120819. doi: 10.1016/j.biomaterials.2021.120819 33892345 PMC10108099

[B19] Ju T. Kong J. Y. Stothard P. Willing B. P. (2019). Defining the Role of Parasutterella, a Previously Uncharacterized Member of the Core Gut Microbiota. ISME J. 13 (6), 1520–1534. doi: 10.1038/s41396-019-0364-5 30742017 PMC6776049

[B20] Kanehisa M. Furumichi M. Tanabe M. Sato Y. Morishima K. (2017). KEGG: New Perspectives on Genomes, Pathways, Diseases and Drugs. Nucleic Acids Res. 45 (D1), D353–d361. doi: 10.1093/nar/gkw1092 27899662 PMC5210567

[B21] Kara F. M. Chitu V. Sloane J. Axelrod M. Fredholm B. B. Stanley E. R. (2010). Adenosine A1 Receptors (A1Rs) Play a Critical Role in Osteoclast Formation and Function. FASEB J. 24 (7), 2325–2333. doi: 10.1096/fj.09-147447 20181934 PMC2887264

[B22] Katebi M. Soleimani M. Cronstein B. N. (2009). Adenosine A2A Receptors Play an Active Role in Mouse Bone Marrow-Derived Mesenchymal Stem Cell Development. J. Leukoc. Biol. 85 (3), 438–444. doi: 10.1189/jlb.0908520 19056861 PMC3059135

[B23] Kwon Y. M. Kim G. W. Yim H. W. Paek Y. J. Lee K. S. (2015). Association Between Dietary Fat Intake and Bone Mineral Density in Korean Adults: Data From Korea National Health and Nutrition Examination Survey IV, (2008 ∼ 2009). Osteoporos. Int. 26 (3), 969–976. doi: 10.1007/s00198-014-2977-x 25491765

[B24] Li Y. Lu L. Xie Y. Chen X. Tian L. Liang Y. (2020). Interleukin-6 Knockout Inhibits Senescence of Bone Mesenchymal Stem Cells in High-Fat Diet-Induced Bone Loss. Front. Endocrinol. (Lausanne) 11, 622950. doi: 10.3389/fendo.2020.622950 33679606 PMC7933660

[B25] Liu W. C. Shyu J. F. Lim P. S. Fang T. C. Lu C. L. Zheng C. M. (2020a). Concentration and Duration of Indoxyl Sulfate Exposure Affects Osteoclastogenesis by Regulating NFATc1 via Aryl Hydrocarbon Receptor. Int. J. Mol. Sci. 21 (10), 3486. doi: 10.3390/ijms21103486 PMC7278944 32429048

[B26] Liu W. C. Shyu J. F. Lin Y. F. Chiu H. W. Lim P. S. Lu C. L. (2020b). Resveratrol Rescue Indoxyl Sulfate-Induced Deterioration of Osteoblastogenesis via the Aryl Hydrocarbon Receptor /MAPK Pathway. Int. J. Mol. Sci. 21 (20), 7483. doi: 10.3390/ijms21207483 PMC7589702 33050571

[B27] Li S. You J. Wang Z. Liu Y. Wang B. Du M. (2021). Curcumin Alleviates High-Fat Diet-Induced Hepatic Steatosis and Obesity in Association With Modulation of Gut Microbiota in Mice. Food Res. Int. 143:110270. doi: 10.1016/j.foodres.2021.110270 33992371

[B28] Lucas S. Omata Y. Hofmann J. Böttcher M. Iljazovic A. Sarter K. (2018). Short-Chain Fatty Acids Regulate Systemic Bone Mass and Protect From Pathological Bone Loss. Nat. Commun. 9 (1), 55. doi: 10.1038/s41467-017-02490-4 29302038 PMC5754356

[B29] Lu L. Chen X. Liu Y. Yu X. (2021). Gut Microbiota and Bone Metabolism. FASEB J. 35 (7), e21740. doi: 10.1096/fj.202100451R 34143911

[B30] Luo Y. Chen G. L. Hannemann N. Ipseiz N. Krönke G. Bäuerle T. (2015). Microbiota From Obese Mice Regulate Hematopoietic Stem Cell Differentiation by Altering the Bone Niche. Cell Metab. 22 (5), 886–894. doi: 10.1016/j.cmet.2015.08.020 26387866

[B31] Madsen K. Cornish A. Soper P. McKaigney C. Jijon H. Yachimec C. (2001). Probiotic Bacteria Enhance Murine and Human Intestinal Epithelial Barrier Function. Gastroenterology 121 (3), 580–591. doi: 10.1053/gast.2001.27224 11522742

[B32] McCabe L. R. Irwin R. Tekalur A. Evans C. Schepper J. D. Parameswaran N. (2019). Exercise Prevents High Fat Diet-Induced Bone Loss, Marrow Adiposity and Dysbiosis in Male Mice. Bone 118, 20–31. doi: 10.1016/j.bone.2018.03.024 29604350 PMC6163087

[B33] McMurdie P. J. Holmes S. (2013). Phyloseq: An R Package for Reproducible Interactive Analysis and Graphics of Microbiome Census Data. PloS One 8 (4), e61217. doi: 10.1371/journal.pone.0061217 23630581 PMC3632530

[B34] Mediero A. Cronstein B. N. (2013). Adenosine and Bone Metabolism. Trends Endocrinol. Metab. 24 (6), 290–300. doi: 10.1016/j.tem.2013.02.001 23499155 PMC3669669

[B35] Mediero A. Kara F. M. Wilder T. Cronstein B. N. (2012). Adenosine A(2A) Receptor Ligation Inhibits Osteoclast Formation. Am. J. Pathol. 180 (2), 775–786. doi: 10.1016/j.ajpath.2011.10.017 22138579 PMC3349861

[B36] Miyamoto T. Hirayama A. Sato Y. Koboyashi T. Katsuyama E. Kanagawa H. (2017). A Serum Metabolomics-Based Profile in Low Bone Mineral Density Postmenopausal Women. Bone 95, 1–4. doi: 10.1016/j.bone.2016.10.027 27989648

[B37] Montalvany-Antonucci C. C. Zicker M. C. Ferreira A. V. M. Macari S. Ramos-Junior E. S. Gomez R. S. (2018). High-Fat Diet Disrupts Bone Remodeling by Inducing Local and Systemic Alterations. J. Nutr. Biochem. 59, 93–103. doi: 10.1016/j.jnutbio.2018.06.006 29986312

[B38] Muhammad S. I. Maznah I. Mahmud R. Zuki A. B. Imam M. U. (2013). Upregulation of Genes Related to Bone Formation by γ-Amino Butyric Acid and γ-Oryzanol in Germinated Brown Rice is via the Activation of GABAB-Receptors and Reduction of Serum IL-6 in Rats. Clin. Interv. Aging 8, 1259–1271. doi: 10.2147/cia.S45943 24098073 PMC3789840

[B39] Noronha-Matos J. B. Correia-de-Sá P. (2016). Mesenchymal Stem Cells Ageing: Targeting the "Purinome" to Promote Osteogenic Differentiation and Bone Repair. J. Cell Physiol. 231 (9), 1852–1861. doi: 10.1002/jcp.25303 26754327

[B40] Ondov B. D. Bergman N. H. Phillippy A. M. (2011). Interactive Metagenomic Visualization in a Web Browser. BMC Bioinformatics 12, 385. doi: 10.1186/1471-2105-12-385 21961884 PMC3190407

[B41] Pepe M. S. Li C. I. Feng Z. (2015). Improving the Quality of Biomarker Discovery Research: The Right Samples and Enough of Them. Cancer Epidemiol. Biomarkers Prev. 24 (6), 944–950. doi: 10.1158/1055-9965.Epi-14-1227 25837819 PMC4452419

[B42] Pouresmaeili F. Kamalidehghan B. Kamarehei M. Goh Y. M. (2018). A Comprehensive Overview on Osteoporosis and its Risk Factors. Ther. Clin. Risk Manag. 14, 2029–2049. doi: 10.2147/tcrm.S138000 30464484 PMC6225907

[B43] Pruesse E. Quast C. Knittel K. Fuchs B. M. Ludwig W. Peplies J. (2007). SILVA: A Comprehensive Online Resource for Quality Checked and Aligned Ribosomal RNA Sequence Data Compatible With ARB. Nucleic Acids Res. 35 (21), 7188–7196. doi: 10.1093/nar/gkm864 17947321 PMC2175337

[B44] Qiao J. Wu Y. Ren Y. (2021). The Impact of a High Fat Diet on Bones: Potential Mechanisms. Food Funct. 12 (3), 963–975. doi: 10.1039/d0fo02664f 33443523

[B45] Qi H. Bao J. An G. Ouyang G. Zhang P. Wang C. (2016). Association Between the Metabolome and Bone Mineral Density in Pre- and Post-Menopausal Chinese Women Using GC-MS. Mol. Biosyst. 12 (7), 2265–2275. doi: 10.1039/c6mb00181e 27168060

[B46] Ridlon J. M. Kang D. J. Hylemon P. B. Bajaj J. S. (2014). Bile Acids and the Gut Microbiome. Curr. Opin. Gastroenterol. 30 (3), 332–338. doi: 10.1097/mog.0000000000000057 24625896 PMC4215539

[B47] Russell J. M. Stephenson G. S. Yellowley C. E. Benton H. P. (2007). Adenosine Inhibition of Lipopolysaccharide-Induced Interleukin-6 Secretion by the Osteoblastic Cell Line MG-63. Calcif. Tissue Int. 81 (4), 316–326. doi: 10.1007/s00223-007-9060-y 17705048

[B48] Scheller E. L. Troiano N. Vanhoutan J. N. Bouxsein M. A. Fretz J. A. Xi Y. (2014). Use of Osmium Tetroxide Staining With Microcomputerized Tomography to Visualize and Quantify Bone Marrow Adipose Tissue *In Vivo* . Methods Enzymol. 537, 123–139. doi: 10.1016/b978-0-12-411619-1.00007-0 24480344 PMC4097010

[B49] Srutkova D. Schwarzer M. Hudcovic T. Zakostelska Z. Drab V. Spanova A. (2015). Bifidobacterium Longum CCM 7952 Promotes Epithelial Barrier Function and Prevents Acute DSS-Induced Colitis in Strictly Strain-Specific Manner. PloS One 10 (7), e0134050. doi: 10.1371/journal.pone.0134050 26218526 PMC4517903

[B50] Styner M. Thompson W. R. Galior K. Uzer G. Wu X. Kadari S. (2014). Bone Marrow Fat Accumulation Accelerated by High Fat Diet Is Suppressed by Exercise. Bone 64, 39–46. doi: 10.1016/j.bone.2014.03.044 24709686 PMC4041820

[B51] Su Y. Elshorbagy A. Turner C. Refsum H. Chan R. Kwok T. (2019). Circulating Amino Acids are Associated With Bone Mineral Density Decline and Ten-Year Major Osteoporotic Fracture Risk in Older Community-Dwelling Adults. Bone 129, 115082. doi: 10.1016/j.bone.2019.115082 31622772 PMC6925590

[B52] Tang M. Lu L. Yu X. (2020). Interleukin-17a Interweaves the Skeletal and Immune Systems. Front. Immunol. 11, 625034. doi: 10.3389/fimmu.2020.625034 33613566 PMC7890031

[B53] van Beek A. A. Fransen F. Meijer B. de Vos P. Knol E. F. Savelkoul H. F. J. (2018). Aged Mice Display Altered Numbers and Phenotype of Basophils, and Bone Marrow-Derived Basophil Activation, With a Limited Role for Aging-Associated Microbiota. Immun. Ageing 15, 32. doi: 10.1186/s12979-018-0135-6 30519273 PMC6263040

[B54] Wang Q. Garrity G. M. Tiedje J. M. Cole J. R. (2007). Naive Bayesian Classifier for Rapid Assignment of rRNA Sequences Into the New Bacterial Taxonomy. Appl. Environ. Microbiol. 73 (16), 5261–5267. doi: 10.1128/aem.00062-07 17586664 PMC1950982

[B55] Wang L. Gong Z. Zhang X. Zhu F. Liu Y. Jin C. (2020). Gut Microbial Bile Acid Metabolite Skews Macrophage Polarization and Contributes to High-Fat Diet-Induced Colonic Inflammation. Gut Microbes 12 (1), 1–20. doi: 10.1080/19490976.2020.1819155 PMC7553752 33006494

[B56] Wang J. Wang Y. Gao W. Wang B. Zhao H. Zeng Y. (2017). Diversity Analysis of Gut Microbiota in Osteoporosis and Osteopenia Patients. PeerJ 5, e3450. doi: 10.7717/peerj.3450 28630804 PMC5474093

[B57] Wen K. Tao L. Tao Z. Meng Y. Zhou S. Chen J. (2020). Fecal and Serum Metabolomic Signatures and Microbial Community Profiling of Postmenopausal Osteoporosis Mice Model. Front. Cell Infect. Microbiol. 10:535310. doi: 10.3389/fcimb.2020.535310 33330117 PMC7728697

[B58] Whisner C. M. Castillo L. F. (2018). Prebiotics, Bone and Mineral Metabolism. Calcif. Tissue Int. 102 (4), 443–479. doi: 10.1007/s00223-017-0339-3 29079996 PMC5851694

[B59] Xie W. Han Y. Li F. Gu X. Su D. Yu W. (2020). Neuropeptide Y1 Receptor Antagonist Alters Gut Microbiota and Alleviates the Ovariectomy-Induced Osteoporosis in Rats. Calcif. Tissue Int. 106 (4), 444–454. doi: 10.1007/s00223-019-00647-5 31844916

[B60] Xing C. Wang M. Ajibade A. A. Tan P. Fu C. Chen L. (2021). Microbiota Regulate Innate Immune Signaling and Protective Immunity Against Cancer. Cell Host Microbe 29 (6), 959–974.e957. doi: 10.1016/j.chom.2021.03.016 33894128 PMC8192480

[B61] You Y. S. Lin C. Y. Liang H. J. Lee S. H. Tsai K. S. Chiou J. M. (2014). Association Between the Metabolome and Low Bone Mineral Density in Taiwanese Women Determined by (1)H NMR Spectroscopy. J. Bone Miner. Res. 29 (1), 212–222. doi: 10.1002/jbmr.2018 23775851

[B62] Zaiss M. M. Jones R. M. Schett G. Pacifici R. (2019). The Gut-Bone Axis: How Bacterial Metabolites Bridge the Distance. J. Clin. Invest. 129 (8), 3018–3028. doi: 10.1172/jci128521 31305265 PMC6668676

[B63] Zhang Z. Lin T. Meng Y. Hu M. Shu L. Jiang H. (2021). FOS/GOS Attenuates High-Fat Diet Induced Bone Loss via Reversing Microbiota Dysbiosis, High Intestinal Permeability and Systemic Inflammation in Mice. Metabolism 119, 154767. doi: 10.1016/j.metabol.2021.154767 33753088

[B64] Zhao L. Qi Z. Yi L. Li J. Cui Y. Ur Rehman F. (2021). The Interaction Between Gut Microbiota and Flavonoid Extract From Smilax Glabra Roxb. And its Potent Alleviation of Fatty Liver. Food Funct. 12 (17), 7836–7850. doi: 10.1039/d1fo00727k 34235516

[B65] Zhao Q. Shen H. Su K. J. Zhang J. G. Tian Q. Zhao L. J. (2018). Metabolomic Profiles Associated With Bone Mineral Density in US Caucasian Women. Nutr. Metab. (Lond.) 15, 57. doi: 10.1186/s12986-018-0296-5 30116286 PMC6086033

[B66] Zhu X. Bai W. Zheng H. (2021). Twelve Years of GWAS Discoveries for Osteoporosis and Related Traits: Advances, Challenges and Applications. Bone Res. 9 (1), 23. doi: 10.1038/s41413-021-00143-3 33927194 PMC8085014

[B67] Zhu X. Zheng H. (2021). Factors Influencing Peak Bone Mass Gain. Front. Med. 15 (1), 53–69. doi: 10.1007/s11684-020-0748-y 32519297

