# Supplementary material for: Gut Microbiota and Serum Metabolic Signatures of High-Fat-Induced Bone Loss in Mice
Source: Front Cell Infect Microbiol. 2021 Dec 22;11:788576. doi: 10.3389/fcimb.2021.788576 (PMC8727351; doi:10.3389/fcimb.2021.788576)
Supplement: Supplementary file 1 [file Table_1.docx]

**Supplementary Table 1. The composition of normal diet (ND) and high fat diet (HFD)**

| **Composition** | **ND (H10010)** | | **HFD (H10060)** | |
| --- | --- | --- | --- | --- |
|  | **Mass ratio (g %)** | **Energy ratio (kcal %)** | **Mass ratio (g %)** | **Energy ratio (kcal %)** |
| Protein | 19.2 | 20 | 26 | 20 |
| Carbohydrate | 67.3 | 70 | 26 | 20 |
| Fat | 4.3 | 10 | 35 | 60 |

**Supplementary Table 2. Primer sequences.**

| **Target gene** | **Forward (5’ to 3’)** | **Reverse (5’ to 3’)** |
| --- | --- | --- |
| β-actin | AGATTACTGCTCTGGCTCCTAGC | ACTCATCGTACTCCTGCTTGCT |
| Col1a1 | CTGGCGGTTCAGGTCCAAT | TTCCAGGCAATCCACGAGC |
| Runx2 | GGTACTTCGTCAGCATCCTATCAG | GCTTCCGTCAGCGTCAACAC |
| Ppar-γ | CACTCGCATTCCTTTGACATC | CGCACTTTGGTATTCTTGGAG |
| Adipoq | ATCTGGAGGTGGGAGACCAA | GGGCTATGGGTAGTTGCAGT |
| Trap | CAGCAGCTCCCTAGAAGATGG | CTGGAACCTCTTGTCGCTGG |
| Ctsk | GAAGAAGACTCACCAGAAGCAG | TCCAGGTTATGGGCAGAGATT |

**Supplementary Table 3. Differential serum metabolites of positive ion mode between ND and HFD group.**

| **Index** | **MS2 name** | **log2 FC(HFD/ND)** | **VIP** | ***P*-value** | **FDR Adjusted *p-*value** |
| --- | --- | --- | --- | --- | --- |
| POS00029 | Deoxyguanosine | -2.9660 | 4.3467 | 0.0007 | 0.0329 |
| POS00039 | Adenosine | -2.7585 | 18.7814 | 0.0003 | 0.0204 |
| POS00054 | 1-(1-Pyrrolidinyl)-2-propanone | -1.5587 | 2.0137 | 0.0010 | 0.0385 |
| POS00085 | gamma-Glutamylisoleucine | 4.0072 | 1.0620 | 0.0005 | 0.0261 |
| POS00214 | Pregabalin | -1.0615 | 9.6906 | 0.0005 | 0.0273 |
| POS00244 | LysoPC(16:1(9Z)/0:0) | -1.3139 | 8.0299 | 0.0000 | 0.0006 |
| POS00269 | 3-Acetamidobutanal | -1.9929 | 4.5439 | 0.0002 | 0.0148 |
| POS00334 | 4-Hydroxy-2-butenoic acid gamma-lactone | -1.1283 | 2.5543 | 0.0001 | 0.0120 |
| POS00398 | PC(16:1(9Z)/16:1(9Z)) | -1.6150 | 2.6378 | < 0.0001 | 0.0064 |
| POS00423 | Rotigotine | 2.1896 | 1.2375 | 0.0001 | 0.0065 |
| POS01464 | N-Formimino-L-aspartate | -1.1447 | 2.9765 | 0.0003 | 0.0206 |
| POS02389 | 2-Aminoethyl diphenylborite | -1.4089 | 2.1194 | 0.0005 | 0.0258 |
| POS02986 | 2-Hydroxymucote semialdehyde | -1.3240 | 4.3380 | 0.0003 | 0.0204 |
| POS04161 | 2,6-Dichloroindophenol | -2.9136 | 6.1700 | 0.0002 | 0.0161 |
| POS04910 | 3,4,5-trihydroxy-6-({15-hydroxy-11,17,18-trimethoxy-13-oxo-6,8,20-trioxapentacyclo[10.8.0.0icosa-1(12),2(9),4,10,14,16,18-heptaen-16-yl}oxy)oxane-2-carboxylic acid | 1.8032 | 1.3471 | 0.0005 | 0.0274 |
| POS05315 | L-dopachromate | 3.8221 | 1.0775 | 0.0004 | 0.0222 |
| POS05326 | 8-Hydroxyluteolin 4'-methyl ether 8-glucoside-3'-sulfate | 1.4167 | 1.0395 | 0.0004 | 0.0222 |

**Supplementary Table 4. Differential serum metabolites of negative ion mode between ND and HFD group.**

| **Index** | **MS2 name** | **log2 FC(HFD/ND)** | **VIP** | **P-value** | **FDR Adjusted *p*-value** |
| --- | --- | --- | --- | --- | --- |
| NEG00013 | Palmitoleic acid | -1.0229 | 13.3562 | 0.0024 | 0.0408 |
| NEG00016 | 11Z-Eicosenoic acid | -1.0038 | 3.8459 | 0.0031 | 0.0490 |
| NEG00073 | 9,10-Epoxyoctadecenoic acid | 2.0024 | 1.0381 | 0.0001 | 0.0035 |
| NEG00127 | Deoxycholic acid | 1.2924 | 1.4131 | 0.0001 | 0.0039 |
| NEG00154 | 1,11-Undecanedicarboxylic acid | 1.8665 | 1.2013 | 0.0002 | 0.0076 |
| NEG03726 | Gallagic acid | -2.3173 | 1.4370 | 0.0000 | 0.0007 |
| NEG04012 | 3',5,6-Trihydroxy-3,4',7,8-tetramethoxyflavone 3-glucoside | 3.4924 | 1.6825 | 0.0000 | 0.0000 |
| NEG04078 | S-(1,2-Dichlorovinyl)thiol | -1.2151 | 1.5641 | 0.0004 | 0.0119 |
| NEG04115 | Irisxanthone | 1.4437 | 1.0140 | 0.0000 | 0.0000 |
| NEG04249 | 3',5,6-Trihydroxy-3,4',7,8-tetramethoxyflavone 3-glucoside | 3.1383 | 1.6443 | 0.0000 | 0.0001 |
| NEG04258 | Kaempferol 7-(6'-galloylglucoside) | 2.6371 | 1.0661 | 0.0000 | 0.0000 |
| NEG04268 | Musca-aurin-II | 1.2377 | 1.8228 | 0.0000 | 0.0001 |
| NEG04294 | N-Acetyl-O-demethylpuromycin-5'-phosphate | 2.5182 | 1.3856 | 0.0000 | 0.0001 |
| NEG04367 | Betanin | 1.8721 | 1.2455 | 0.0020 | 0.0364 |
| NEG04375 | Cyanidin 3-O-(beta-D-xylosyl-(1-&gt;2)-beta-D-galactoside) | 6.1460 | 1.2288 | 0.0000 | 0.0000 |
| NEG04444 | Cyclochlorotine | 1.4449 | 1.3822 | 0.0006 | 0.0157 |
| NEG04474 | 8-Hydroxyluteolin 4'-methyl ether 8-glucoside-3'-sulfate | 1.7196 | 1.4727 | 0.0000 | 0.0001 |
| NEG04481 | Irisxanthone | 1.5018 | 1.9991 | 0.0001 | 0.0051 |
| NEG04488 | (2S,3R)-2-[(1R)-1-[3,5-bis(trifluoromethyl)phenyl]ethoxy]-3-(4-fluorophenyl)morpholine | 2.2955 | 1.2728 | 0.0000 | 0.0001 |
| NEG04624 | 3,4,5-trihydroxy-6-({15-hydroxy-11,17,18-trimethoxy-13-oxo-6,8,20-trioxapentacyclo[10.8.0.0icosa-1(12),2(9),4,10,14,16,18-heptaen-16-yl}oxy)oxane-2-carboxylic acid | 1.6316 | 1.6417 | 0.0000 | 0.0020 |
| NEG04705 | 3,4,5-trihydroxy-6-[2-hydroxy-3-(7-hydroxy-4-oxo-4H-chromen-2-yl)-6-methoxyphenoxy]oxane-2-carboxylic acid | 1.1798 | 1.4778 | 0.0029 | 0.0471 |
| NEG04754 | BL V | 2.3258 | 2.9058 | 0.0001 | 0.0036 |
| NEG04769 | 2-(2,5-dihydroxy-4-methoxyphenyl)-5,7-dihydroxy-6-[3,4,5-trihydroxy-6-(hydroxymethyl)oxan-2-yl]-4H-chromen-4-one | 1.1310 | 1.2095 | 0.0002 | 0.0062 |
| NEG04921 | Musca-aurin-II | 1.4989 | 2.3007 | 0.0003 | 0.0099 |
| NEG04943 | Ponceau MX | 1.9113 | 1.7868 | 0.0000 | 0.0006 |
| NEG04982 | Epitheaflavic acid 3'-gallate | 1.7278 | 1.8229 | 0.0000 | 0.0000 |
| NEG04999 | 8-Hydroxyluteolin 4'-methyl ether 8-glucoside-3'-sulfate | 1.6528 | 4.7287 | 0.0000 | 0.0001 |
| NEG05000 | Celecoxib glucuronide | 1.5259 | 2.8163 | 0.0000 | 0.0001 |
| NEG05002 | Ketoconazole | 1.2307 | 1.3011 | 0.0001 | 0.0039 |
| NEG05003 | 3,4,5-trihydroxy-6-({6-[3-(4-methoxy-1-benzofuran-5-yl)-3-oxopropanoyl]-2H-1,3-benzodioxol-5-yl}oxy)oxane-2-carboxylic acid | 1.4191 | 2.3049 | 0.0000 | 0.0001 |
| NEG05008 | {2-[5,7-dihydroxy-2-(3-methoxyphenyl)-4-oxo-4H-chromen-6-yl]-3-hydroxy-6-methyl-5-oxooxan-4-yl}oxidanesulfonic acid | 2.3602 | 2.8964 | 0.0000 | 0.0000 |
| NEG05009 | Dide-O-methyl-4-O-alpha-D-glucopyranosylsimmondsin | 2.7242 | 1.7186 | 0.0000 | 0.0000 |
| NEG05011 | Biochanin A 7-O-(6-O-malonyl-beta-D-glucoside) | 1.3066 | 2.8563 | 0.0000 | 0.0000 |
| NEG05039 | 1,3,5,8-Tetrahydroxy-6-methoxy-2-methylanthraquinone 8-O-b-D-glucoside | 1.0827 | 1.3652 | 0.0000 | 0.0004 |
| NEG05077 | Chicoric acid | 1.0082 | 1.4160 | 0.0004 | 0.0124 |
| NEG05087 | 3,4,5-trihydroxy-6-({3-[3-methoxy-4-(sulfooxy)phenyl]prop-2-enoyl}oxy)oxane-2-carboxylic acid | 1.5030 | 2.0303 | 0.0007 | 0.0183 |
| NEG05092 | Salviaflaside methyl ester | 2.1470 | 1.7182 | 0.0000 | 0.0001 |
| NEG05094 | BL V | 2.1679 | 2.1252 | 0.0000 | 0.0000 |
| NEG05101 | 3,4,5-trihydroxy-6-{[3-(4-methoxy-1-benzofuran-5-yl)-3-oxopropanoyl]oxy}oxane-2-carboxylic acid | 2.2202 | 1.7354 | 0.0000 | 0.0000 |
| NEG05102 | Mangiferin | 1.5391 | 1.4896 | 0.0000 | 0.0012 |
| NEG05103 | 6-Hydroxyluteolin 6-xyloside | 2.4957 | 1.1442 | 0.0000 | 0.0000 |
| NEG05105 | C.I. Pigment Blue 63 | 1.0057 | 1.3811 | 0.0001 | 0.0025 |
| NEG05123 | Loquatoside | 4.8067 | 1.6583 | 0.0000 | 0.0000 |
| NEG05148 | Irisxanthone | 2.7821 | 1.2529 | 0.0000 | 0.0000 |
| NEG05424 | N-(6-Oxo-6H-dibenzo[b,d]pyran-3-yl)maleamic acid | -1.3041 | 2.2062 | 0.0005 | 0.0135 |
| NEG05495 | 3,4-dihydroxy-5-[2,4,5-trihydroxy-3-(3,4,5-trihydroxybenzoyloxy)benzoyloxy]benzoic acid | 1.3999 | 1.3529 | 0.0001 | 0.0024 |
| NEG05618 | L-Threonylcarbamoyladenylate | -2.4688 | 2.5244 | 0.0022 | 0.0386 |

**Supplementary Table 5. The details of ND-vs-HFD Pathway Enrichment**

| # | **Pathway** | **Pathway ID** | **C_id** | **Differentially expressed metabolites** | **MS2 name** |
| --- | --- | --- | --- | --- | --- |
| 1 | Purine metabolism | Ko00230 | C00212 | POS00039 | Adenosine |
|  |  |  | C00330 | POS00029 | Deoxyguanosine;  2'-Deoxyguanosine |
| 2 | [Regulation of lipolysis in adipocyte](file:///E:\lly\内分泌实验室\article%20菌群和高脂饮食\投稿\Frontiers\最新关联分析\GHDM191469-1_sup_10\result\1.diff_enrich\POS\enrich\KO\ND-vs-HFD_map\map04923.html) | ko04923 | C00212 | POS00039 | Adenosine |
| 3 | [cGMP - PKG signaling pathway](file:///E:\lly\内分泌实验室\article%20菌群和高脂饮食\投稿\Frontiers\最新关联分析\GHDM191469-1_sup_10\result\1.diff_enrich\POS\enrich\KO\ND-vs-HFD_map\map04022.html) | ko04022 | C00212 | POS00039 | Adenosine |
| 4 | [Renin secretion](file:///E:\lly\内分泌实验室\article%20菌群和高脂饮食\投稿\Frontiers\最新关联分析\GHDM191469-1_sup_10\result\1.diff_enrich\POS\enrich\KO\ND-vs-HFD_map\map04924.html) | ko04924 | C00212 | POS00039 | Adenosine |
| 5 | [Morphine addiction](file:///E:\lly\内分泌实验室\article%20菌群和高脂饮食\投稿\Frontiers\最新关联分析\GHDM191469-1_sup_10\result\1.diff_enrich\POS\enrich\KO\ND-vs-HFD_map\map05032.html) | ko05032 | C00212 | POS00039 | Adenosine |


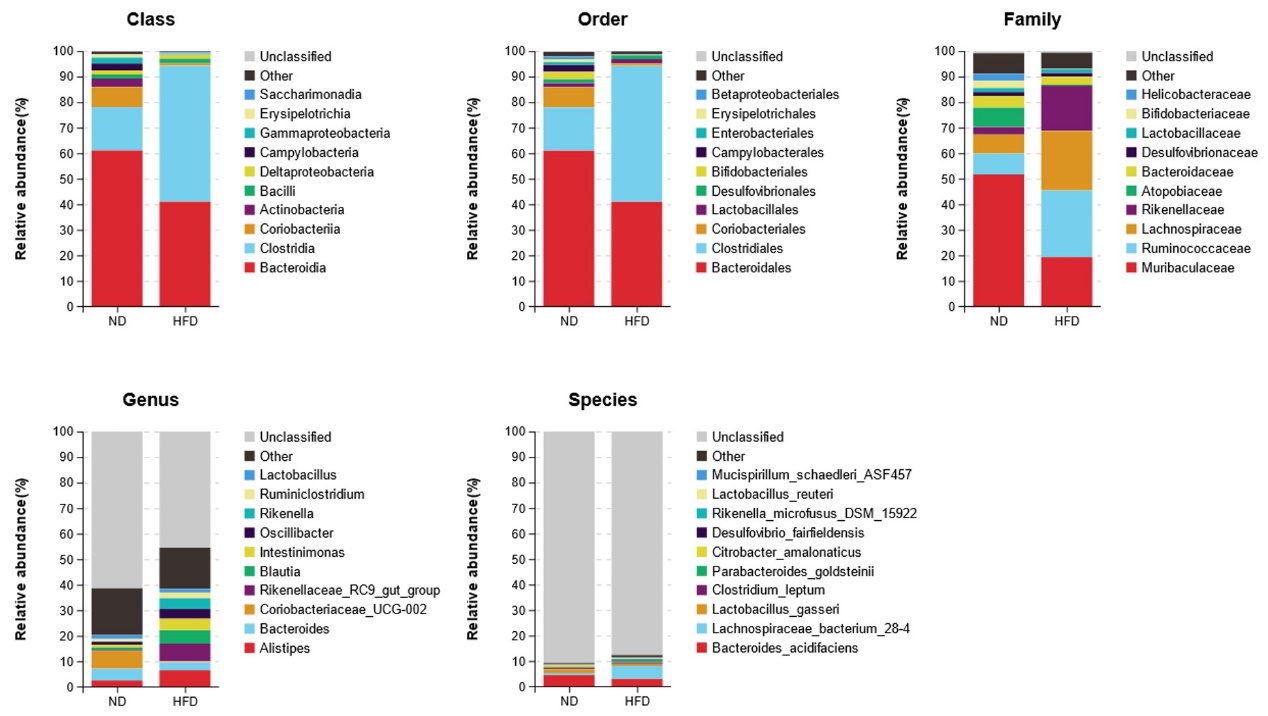


**Supplementary Figure 1. Composition analysis of gut microbiota at a class, order, family, genus and species level.**


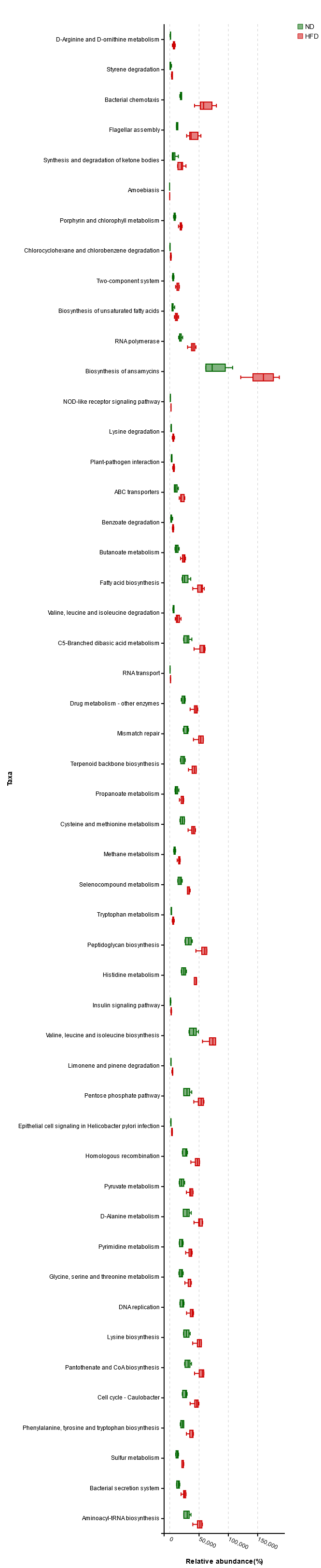

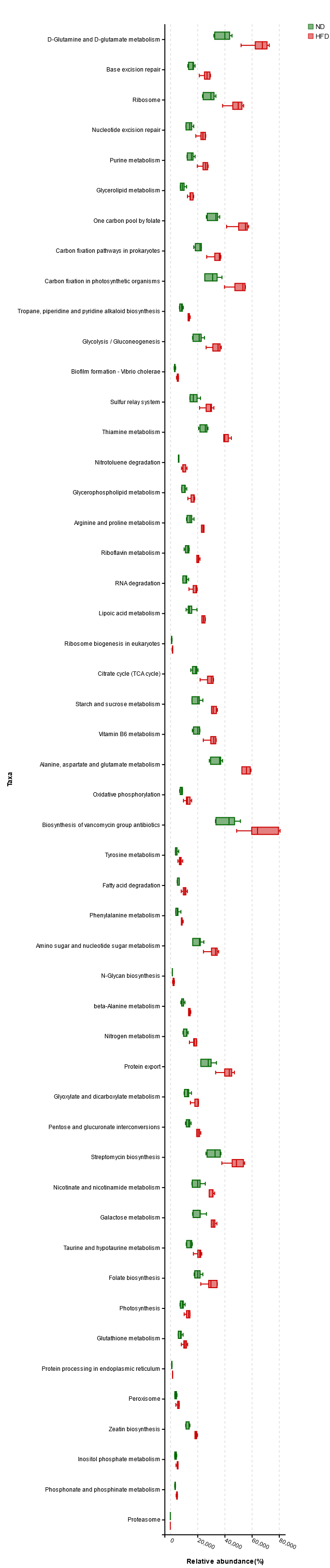


**Supplementary Figure 2. Analysis of the enriched KEGG pathway in level 3 (*P* < 0.01)**

**
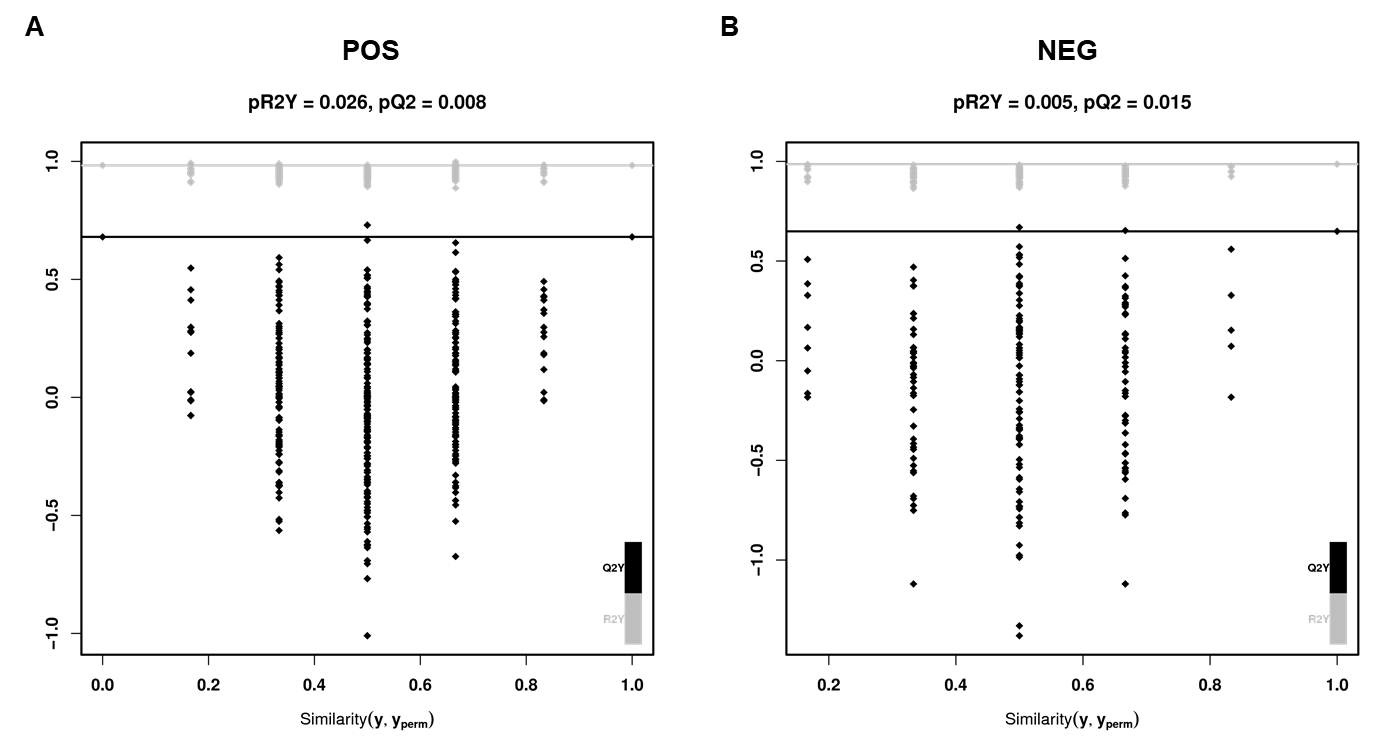
**

**Supplementary Figure 3. Permutation tests of OPLS-DA for metabolites in POS and NEG modes.** POS, positive ion modes; NEG, negative ion modes.

**
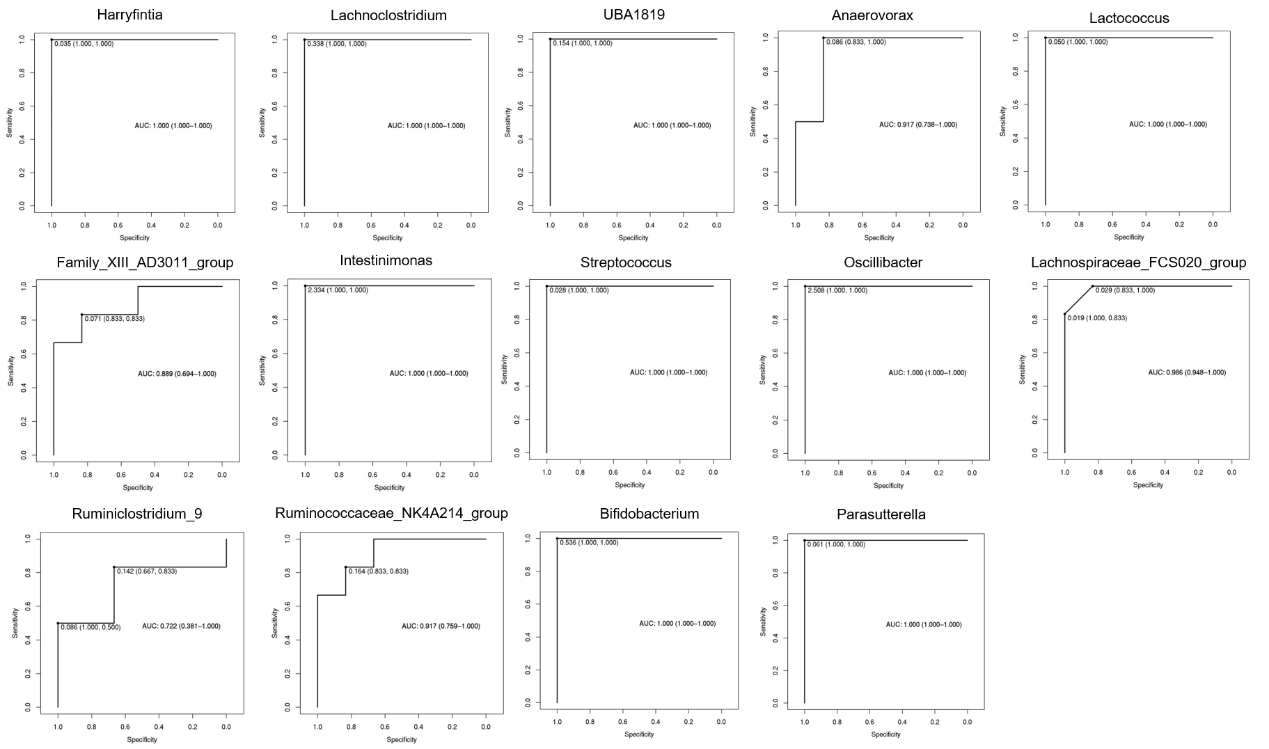
**

**Supplementary Figure 4.** **ROC curves of some differential bacteria correlated to BV/TV (|r| ≥ 0.8)**

**
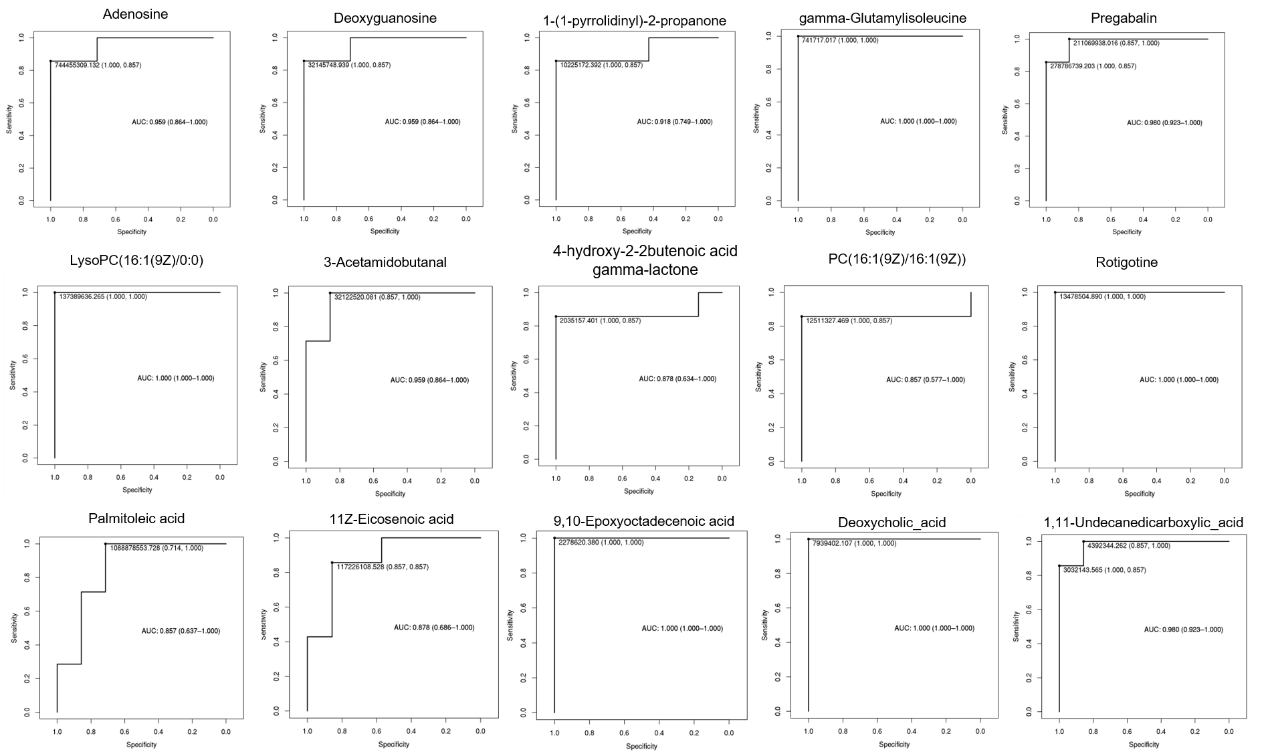
**

**Supplementary Figure 5.** **ROC curves of some differential metabolites correlated to BV/TV (|r| ≥ 0.6)**
